# Resuscitating Through the Smoke: Novel Use of Cyanocobalamin in a Pediatric House Fire Victim

**DOI:** 10.7759/cureus.83500

**Published:** 2025-05-05

**Authors:** David R Janese, Michael Donald, Emily McGee, Jacquelyn Bowers

**Affiliations:** 1 Emergency Medicine/Family Medicine, Louisiana State University Health Shreveport (LSUHS), Shreveport, USA; 2 Pharmacology, Louisiana State University Health Shreveport (LSUHS), Shreveport, USA; 3 Emergency Medicine, Louisiana State University Health Shreveport (LSUHS), Shreveport, USA

**Keywords:** carbon monoxide (co) poisoning, cyanide poisoning, hydroxocobalamin, pediatric resuscitation, push-dose medication administration, smoke inhalation injury

## Abstract

Hydroxocobalamin (Cyanokit® (HealthTech BioActives, Spain)) is the first-line antidote for suspected cyanide toxicity, typically infused intravenously over 10-15 minutes. However, in critically unstable patients, faster delivery may be essential. This case describes a seven-year-old female patient found pulseless after exposure to heavy smoke in a residential fire. Following return of spontaneous circulation (ROSC) in the emergency department, the patient remained severely acidotic and hypoxic despite confirmation of a secure airway and initiation of mechanical ventilation. A 5 g dose of hydroxocobalamin was administered as a rapid push over two minutes. The intervention was well tolerated and associated with a significant 13.8% decrease in carboxyhemoglobin levels within 20 minutes, alongside progressive improvement in metabolic parameters. Although the patient ultimately suffered irreversible hypoxic-ischemic brain injury, this case highlights the potential utility of push-dose hydroxocobalamin in time-critical resuscitation scenarios involving suspected cyanide toxicity.

## Introduction

Cyanide toxicity is a rare but life-threatening complication of smoke inhalation in enclosed-space structural fires. It results from the combustion of synthetic materials, such as polyurethane, wool, and plastics, that release hydrogen cyanide gas. Once inhaled, cyanide disrupts oxidative phosphorylation by inhibiting cytochrome oxidase, leading to mitochondrial dysfunction and profound cellular hypoxia. This toxicity often occurs in conjunction with carbon monoxide (CO) poisoning, a dual exposure that can precipitate rapid cardiovascular collapse, metabolic acidosis, and death [1]. In pediatric patients, the risk is exacerbated due to their higher metabolic rates and smaller physiologic reserves, making early recognition and treatment essential [2].

Hydroxocobalamin, a vitamin B₁₂ analog marketed as Cyanokit® (HealthTech BioActives, Spain), is the first-line antidote for cyanide toxicity. It binds free cyanide ions to form cyanocobalamin, a non-toxic compound that is eliminated renally [3,4]. The FDA recommends intravenous (IV) infusion of 5 g over 15 minutes in adults and 70 mg/kg in pediatric patients. However, this delivery may be insufficient in critically ill individuals where rapid reversal of cellular hypoxia is needed. Whether this method can be safely translated to pediatric resuscitation remains uncertain.

To our knowledge, no published pediatric cases describe the use of push-dose hydroxocobalamin during active resuscitation. This report aims to fill that gap by describing the novel use of push-dose hydroxocobalamin in a seven-year-old female in cardiac arrest due to smoke inhalation. The case illustrates the potential utility and safety of this expedited antidote administration strategy in time-critical resuscitative efforts involving suspected cyanide toxicity.

## Case presentation

Patient information

A seven-year-old female was extricated from an active residential structure fire after a prolonged and unspecified duration of heavy smoke exposure. She was found unresponsive and pulseless at the scene. Emergency Medical Services (EMS) initiated advanced life support and transported her directly to the emergency department (ED) while resuscitative efforts were ongoing. According to EMS personnel, she was exposed to dense smoke in a closed-space fire, with no available information regarding burn injuries or trauma.

During transport, the patient received six doses of 0.35 mg epinephrine (1:10,000) and a cumulative 35 mEq of 8.4% sodium bicarbonate via intraosseous (IO) access. While this dosing may appear high, it reflects prolonged cardiopulmonary resuscitation in the field for a presumed toxic-metabolic arrest, consistent with Pediatric Advanced Life Support (PALS) guidelines, which allow repeat epinephrine dosing every three to five minutes.

Upon ED arrival, the patient remained in cardiopulmonary arrest with high-quality chest compressions in progress. Initial airway management by EMS included placement of an iGel supraglottic airway and bag-valve mask ventilation. On exam, she had a Glasgow Coma Scale (GCS) score of 3 and no palpable pulses. Physical examination noted erythematous lips and bilateral conjunctival injection. Despite her pulseless status, her skin appeared warm and pink - an appearance sometimes observed in cyanide toxicity due to impaired tissue oxygen extraction, resulting in misleadingly preserved peripheral perfusion.

Initial arterial blood gas analysis revealed profound metabolic and respiratory acidosis: pH 6.745 (normal 7.35-7.45), pCO₂ 118 mmHg (normal 35-45 mmHg), and pO₂ 31.5 mmHg (normal 80-100 mmHg). Lactate was markedly elevated at 16 mmol/L (normal <2 mmol/L), consistent with severe anaerobic metabolism. The carboxyhemoglobin (COHb) level was critically high at 51.5% (normal <2%, levels >25% are considered severe and life-threatening), and methemoglobin was 2.0% (normal <1.5%). Bicarbonate was severely depleted at 8.2 mEq/L (normal 22-28 mEq/L), reflecting profound metabolic derangement.

Following administration of a 5 g dose of hydroxocobalamin (Cyanokit®) via IV push over approximately two minutes, repeat laboratory tests at 10 minutes showed minimal pH improvement (6.764), worsening hypercapnia (pCO₂ 131 mmHg), and a COHb level of 37.5%, with methemoglobin rising to 8.2%. However, at 20 minutes post-administration, the arterial blood gas showed marked improvement: pH 7.104, pCO₂ 83.5 mmHg, pO₂ 262.1 mmHg, COHb 37.7%, methemoglobin 6.9%, and bicarbonate 20 mEq/L.

These laboratory results strongly supported a diagnosis of mixed CO and cyanide poisoning. Notably, methemoglobin increased from 2.0% to 8.2% post-treatment-likely attributable to hydroxocobalamin itself, which has been documented to cause dose-related elevations in methemoglobin as part of its oxidative mechanism.

Therapeutic interventions

Upon arrival in the emergency department, IO access was already in place; however, it was replaced to optimize fluid and medication delivery. A 22-gauge peripheral IV line was established and used for administration of medications, including the 5-g dose of hydroxocobalamin, which was delivered as a push-dose over approximately two minutes due to the patient’s profound hemodynamic instability. A femoral central venous catheter was subsequently inserted during the ongoing stabilization process.

To address the patient’s combined metabolic and respiratory acidosis, escalating doses of sodium bicarbonate were administered in a tiered fashion, ranging from 1 to 3 mEq/kg. Push-dose epinephrine at a dose of 20 mcg was also administered, followed by initiation of a continuous norepinephrine infusion at 0.02 mcg/kg/min, which was titrated to 0.05 mcg/kg/min to maintain a mean arterial pressure above 65 mmHg.

Airway management was escalated by exchanging the iGel supraglottic airway for an endotracheal tube. Due to the urgency of the clinical situation, neither rapid nor delayed sequence intubation was performed. Mechanical ventilation was initiated using assist control-volume control (ACVC) mode. Initial ventilator settings included a tidal volume of 5 mL/kg, a positive end-expiratory pressure (PEEP) of 5 cm H₂O, and a respiratory rate of 30 breaths per minute. These parameters were later adjusted to a tidal volume of 4 mL/kg, PEEP of 12.5 cm H₂O, and respiratory rate of 60 with a 1:1 inspiratory-to-expiratory ratio in order to address persistent hypercapnia and enhance CO₂ clearance.

Volume resuscitation was initiated using Lactated Ringer’s solution delivered via a push-pull syringe technique at a dose of 20 mL/kg to treat presumed distributive and metabolic shock.

Response to treatment

The patient achieved return of spontaneous circulation (ROSC) after two cycles of cardiopulmonary resuscitation in the emergency department. Following initial stabilization and administration of hydroxocobalamin, she experienced a second cardiac arrest that coincided with worsening hypercapnia (pCO₂ 131 mmHg). In response, ventilator settings were rapidly adjusted to a more aggressive lung-protective strategy optimized for CO₂ clearance. These modifications included an increased respiratory rate, higher PEEP, and an altered inspiratory-to-expiratory ratio. The intervention was successful, and the patient again regained spontaneous circulation.

Serial laboratory analyses demonstrated progressive metabolic improvement following antidote administration and ventilatory optimization. Arterial blood gases showed rising pH and bicarbonate levels, accompanied by a steady decline in COHb concentrations - findings consistent with enhanced oxygen utilization and early mitochondrial recovery.

In the hours following hydroxocobalamin administration, the patient’s urine was noted to have a deep red discoloration, a well-documented and benign effect of the drug. This chromaturia, caused by renal excretion of the red-colored cobalamin compound, can persist for up to several days and may interfere with colorimetric laboratory assays, although no such interference was observed in this case.

Follow-up and outcomes

After resuscitation in the emergency department, the patient was admitted to the pediatric intensive care unit (PICU) for ongoing multi-organ support. Extracorporeal membrane oxygenation (ECMO) was considered during the initial phase of management; however, the critical care team determined that the patient was not a suitable candidate due to diminished cardiac function and persistently elevated COHb levels. Throughout her admission, renal function remained within normal limits, and urinary output was preserved despite aggressive fluid and pharmacologic interventions. No evidence of acute kidney injury was documented.

Despite initial improvements in hemodynamic and metabolic parameters, the patient failed to demonstrate any signs of neurologic recovery. Serial neurologic examinations, along with confirmatory ancillary testing, revealed the absence of brain activity. Following multidisciplinary discussions and careful communication with the patient’s family, the decision was made to compassionately withdraw life-sustaining therapy approximately 48 hours after admission. The family was fully engaged throughout the process, and although they were initially hopeful, they ultimately concurred with the medical team’s assessment that continued care would not be beneficial.

## Discussion

Cyanide toxicity is a fatal condition that may occur with smoke inhalation in enclosed-space fires, especially when synthetic materials like plastics are burning. In pediatric victims, the risk is elevated because of a higher metabolic rate and smaller physiologic reserves, making early recognition and treatment vital [2]. This patient encounter highlighted the successful use of hydroxocobalamin in a critically ill pediatric patient with elevated lactic acidosis, hypercapnia, and elevated COHb, suggesting concurrent cyanide and CO toxicity.

Hydroxocobalamin, the active ingredient in Cyanokit®, is the first-line antidote for cyanide poisoning. It acts by binding free cyanide ions to form cyanocobalamin, which is excreted renally and is non-toxic. These complexes, known as free cobalamins, are used to assess the active drug concentration in the body. Pharmacokinetic studies have shown that higher doses of Cyanokit result in dose-proportional increases in free and total cobalamin levels, with the drug remaining active in the body for approximately 26 to 31 hours. This prolonged half-life and rapid rise in cobalamin concentrations support the use of a push dose in cardiac arrest, as it quickly achieves therapeutic levels, potentially improving hemodynamic response and reversing cyanide toxicity more effectively [3]. The FDA-recommended adult dose is 5 g administered intravenously over 15 minutes, with pediatric dosing at 70 mg/kg (not to exceed 5 g per dose) [4]. This rapid infusion is intended to deliver the antidote swiftly, while avoiding adverse effects such as hypertension, allergic reactions, or acute renal failure [5-7].

In this case, the patient was in active cardiac arrest, with a pH of 6.745 and COHb of 51.5%, indicating severe mitochondrial dysfunction. Upon ROSC, due to the critical nature of the arrest and the suspected cyanide burden, hydroxocobalamin was administered as a push-dose over approximately two minutes, rather than the standard 15-minute infusion. While this administration route is not described in FDA guidance, the urgency of cellular recovery during resuscitation justified deviation from standard protocol. Hydroxocobalamin (Cyanokit®) is typically administered intravenously using the provided infusion set, which includes an in-line filter to remove particulate matter. In our case, due to the patient’s critical condition and need for rapid intervention, the push-dose was administered without the filter to expedite drug delivery. While this bypassed filtration and carried a theoretical risk of particulate infusion, the immediate need to reverse cyanide toxicity and support hemodynamic collapse outweighed the potential risk.

Clinical improvement was notable following this intervention. The patient’s COHb level decreased from 51.5% to 37.7% within 20 minutes, accompanied by rising pH and bicarbonate levels in line with the sodium bicarbonate boluses. These findings suggest not only enhanced oxygen delivery but also reversal of mitochondrial inhibition consistent with cyanide clearance [4,7]. In addition to its role in reversing cyanide toxicity, hydroxocobalamin has shown promise in treating vasodilatory shock by scavenging nitric oxide and hydrogen sulfide, leading to improved vascular tone and increased mean arterial pressure. Studies in adults have demonstrated reduced vasopressor requirements following a 5-g IV dose, suggesting potential benefits in shock states beyond cyanide toxicity. In our pediatric patient, who experienced cardiac arrest and refractory shock, the push-dose administration of hydroxocobalamin may have contributed to ROSC through similar hemodynamic mechanisms [8].

The literature strongly supports rapid hydroxocobalamin administration in cases of suspected cyanide poisoning. In a prospective study of 69 patients with smoke inhalation, Borron et al. reported a 72% survival rate, with 67% of confirmed cyanide-toxic patients surviving after hydroxocobalamin treatment delivered as a rapid infusion [9]. Time-to-antidote delivery was considered a critical factor in these outcomes. Additionally, a case report by Zakharov et al. demonstrated full neurologic recovery after sequential hydroxocobalamin doses were administered over 25 minutes in a patient with confirmed cyanide ingestion/inhalation and lactic acidosis [10].

Though no published data exist on pediatric push-dose administration, adult toxicologic studies suggest that hydroxocobalamin is well tolerated even at high doses and can be administered rapidly without major complications [3,5,6]. Importantly, no adverse hemodynamic effects or allergic reactions occurred in our case, reinforcing the potential safety of this off-label approach when faced with imminent cardiovascular collapse. The rapid absorption rate of inhaled hydrogen cyanide necessitates swift intervention; administering hydroxocobalamin slowly may limit its effectiveness in these cases. Therefore, in our patient's critical condition, administering a push-dose of hydroxocobalamin was a strategic decision to promptly counteract cyanide toxicity and restore spontaneous circulation [5].

This case also underscores the challenges in managing combined cyanide and CO poisoning, particularly in pediatric patients who may present in extremis. While the eventual outcome was poor due to severe hypoxic-ischemic brain injury, the case highlights a critical learning point: in time-sensitive toxicologic emergencies, standard infusion protocols may be insufficient, and push-dose antidotal therapy may offer life-saving benefits when appropriately applied.

Further research is warranted to examine the pharmacokinetics, outcomes, and safety of rapid bolus hydroxocobalamin administration, especially in pediatric resuscitation scenarios. Until such data are available, this case suggests that clinical judgment in critical care scenarios may justify deviation from traditional dosing strategies when immediate reversal of cellular toxicity is essential.

## Conclusions

This case illustrates the potential efficacy and safety of push-dose hydroxocobalamin in the resuscitation of a seven-year-old female patient in cardiac arrest due to smoke inhalation and suspected cyanide toxicity. While traditionally infused over 15 minutes, hydroxocobalamin may be administered as a rapid bolus in critically unstable patients when immediate reversal of cellular hypoxia is essential. The observed reduction in COHb levels and improvement in metabolic parameters support its use in time-sensitive toxicologic emergencies. Cyanide toxicity should be strongly suspected in fire victims with lactic acidosis, cardiac arrest, or altered mental status, particularly when CO exposure is also present. Empirical treatment should not be delayed for laboratory confirmation, and emergency clinicians must be prepared to modify standard protocols based on patient acuity and real-time clinical judgment. As such, clinical judgment remains a cornerstone of successful critical care.
